# IL-13 -1112 polymorphism and periodontitis susceptibility: a meta-analysis

**DOI:** 10.1186/s12903-018-0481-y

**Published:** 2018-02-07

**Authors:** Wenbo Zhang, Pu Xu, Zhuogeng Chen, Yanan Cheng, Xiaoni Li, Qiuhua Mao

**Affiliations:** Department of Oral Implantation, Affiliated Haikou Hospital,Xiangya Medical School, Central South University, Hainan Provincial Stomatology Centre, Haikou, 570208 Hainan Province China

**Keywords:** Periodontitis, Gene polymorphism, Interleukin-13, Meta-analysis

## Abstract

**Background:**

Several studies have examined the association between the IL-13 -1112C/T polymorphism and the risk of periodontitis. However, these studies have reached different conclusions. The aim of the current study was to investigate the link between this IL-13 -1112 polymorphism and susceptibility to periodontitis.

**Methods:**

We utilized electronic databases, including the CNKI (China National Knowledge Infrastructure), Wanfang, PubMed, Embase, and Cochrane Library databases, to manually search for relevant research published through November 30, 2016. The Chinese and English terms used to search the literature included “periodontitis”, “periodontal disease”, “IL 13”, “IL-13”, and “interleukin-13”. In accordance with our inclusion criteria, we selected studies that involved case-control trials. All of these case-control trials described their objectives, design and specific statistical methods. For all included studies, odds ratios (ORs) and 95% confidence intervals (95% CIs) were provided or could be calculated from the study data. The quality of the included literature was evaluated using the Newcastle-Ottawa scale (NOS). STATA 12.0 was used to calculate the sizes of the combined effects and conduct a sensitivity analysis of the results.

**Results:**

Our meta-analysis included 4 articles representing 5 case-control studies with a total of 710 cases and 671 control subjects. The meta-analysis results indicated that the CC vs TT model, CT vs TT model and TT vs CT + CC model (CC VS TT: OR = 0.615, 95% CI = 0.395–0.957; CT vs TT: OR = 0.518, 95% CI = 0.323–0.830; and TT vs CT + CC: OR = 1.739, 95% CI = 1.130–2.676) were significant in five IL-13 -1112 gene polymorphism and periodontitis susceptibility models. Subgroup analysis indicated that the CC vs TT, CT vs TT and TT vs CT + CC models were significant in the chronic periodontitis (CP) group, whereas no significant differences were found in the five aggressive periodontitis (AgP) group models. The sensitivity analysis showed that dropping any single study did not affect the pooled analysis results.

**Conclusion:**

The IL-13 -1112 polymorphism may be associated with susceptibility to periodontitis. The IL-13 -1112 gene polymorphism may be associated with susceptibility to CP but not to AgP. Thus, large-scale, multi-ethnic case-control trials are still warranted.

## Background

Periodontitis is a chronic infectious disease with a high incidence that is amajor cause of tooth loss. Periodontitis is classified into two major types: chronic periodontitis (CP) and aggressive periodontitis (AgP) [1, 2]. Periodontitis is also recognized as one of the most common diseases worldwide, with a prevalence of 15–20% [3]. Research has indicated that the occurrence of periodontitis is the result of multiple factors. Geneticvariation may be a factor that contributes to individual susceptibility to periodontitis [4]. Recent research has indicated that polymorphisms in multiple genes are associated with susceptibility to periodontitis [5, 6]. Interleukin-13 (IL-13) is a pleiotropic cytokine produced by type 2 helper T (TH2) lymphocytes. Single nucleotide polymorphisms (SNPs) have been identified in the interleukin (IL)-13 gene, including two SNPs (−1512A/C and -1112C-T) in the 5′-terminal regulatory region and one SNP (+1923C–T) in the third intron [7]. These SNPs have extremely complex biological effects and are involved in various physiological processes; additionally, they can exhibit different effects under different conditions and can even play dual roles in the same pathophysiological process. The association between the IL-13 -1112 gene polymorphism and the susceptibility to periodontitis has been studied recently. However, inconsistencies exist in the conclusions due to the limited sample sizes and possible differences between studies. These studies are based on small samples in case-control studies; thus, the credibility of the conclusions has been questioned. Therefore, the IL-13 -1112 gene polymorphism and its possible correlation with periodontitis must be analyzed from an evidence-based medicine perspective. A meta-analysis is a combination of multiple effects of the same purpose to overcome the shortcomings of small sample studies by performing a comprehensive analysis and objectively evaluating the statistical methods. This approach can obtain more credible conclusions than a single study. Recently, meta-analyses have been widely used in the field of genetic polymorphism research. The aim of this study was to use a meta-analysis to evaluate the relationship between the IL-13-1112C/T polymorphism and susceptibility to periodontal disease.

## Methods

### Search strategy

Data were collected from the CNKI (China National Knowledge Infrastructure), Wanfang, PubMed (PubMed is an interface for the Medline database), Embase and Cochrane Library databases. Published articles in Chinese and English that addressed the IL-13-1112C/T SNP and susceptibility to periodontitis were retrieved. The retrieval process involved the use of Chinese and English keywords, including “periodontitis”, “periodontal disease”, “IL 13”, “IL-13”, and “interleukin-13”. Relevant publications were also identified via searches supplemented by a literature review. The meta-analysis was restricted to articles published on or before November 30,2016.The search strategy is asfollows (Table 1):

**Table 1:** Search strategy

PubMed	Embase	Cochrane Library	CNKI	WanFang
Search (((“Periodontitis”[Mesh]) OR (((Periodontitides[Title/Abstract]) OR Pericementitis[Title/Abstract]) OR Pericementitides[Title/Abstract]))) AND ((“Interleukin-13”[Mesh]) OR ((((IL-13[Title/Abstract]) OR Interleukin 13[Title/Abstract]) OR IL 13[Title/Abstract]) OR IL13[Title/Abstract]))	‘periodontitis’/exp. OR ‘periodontitides’:ti,ab OR ‘pericementitis’:ti,ab OR ‘pericementitides’:ti,ab AND (‘interleukin 13’/exp. OR ‘il-13’:ti,ab OR ‘interleukin-13’:ti,ab OR ‘il 13’:ti,ab OR ‘il13’:ti,ab)	#1:MeSH descriptor: [Periodontitis] explode all trees #2:Periodontitides:ti,ab,kw or Pericementitis:ti,ab,kw or Pericementitides:ti,ab,kw (Word variations have been searched) #3: #1 or #2 #4:MeSH descriptor: [Interleukin-13] explode all trees #5: IL-13:ti,ab,kw or Interleukin 13:ti,ab,kw or IL 13:ti,ab,kw or IL13:ti,ab,kw (Word variations have been searched) #6: #4 or #5	Subject = Periodontitis or Topic = Periodontal Disease and Subject = Interleukin 13 or Subject = IL-13 or Subject = IL 13 or Subject = Interleukin-13 (Exact Match)	Subject: (periodontitis + periodontal disease) * Subject: (interleukin 13+ IL-13 + IL 13+ Interleukin-13) *

### Conditions for study inclusion

All included studieswere required to satisfy the following three conditions.

Inclusion: 1. The literature was limited to domestic and foreign published cases of periodontal disease associated with the IL-13 -1112C/T gene polymorphism in Chinese and English; 2. The case group of patients had a clinical diagnosis of periodontitis, including CP patients and AgP patients, whereas the control group was the periodontal healthy population; additionally, the case and control groups were not associated with other systemic diseases; 3. Indicators were provided in the study, including the IL-13-1112C/T odds ratio (OR) and the 95% confidence interval (95% CI), for the genotype frequency and the allele frequency, or the original data provided were sufficient to perform the calculations; and 4. When multiple document data were identical or overlapping, the most recently published literature was selected.

Exclusion: 1. Non-genetic polymorphism research was excluded; 2. Polymorphic loci genotype distribution frequencies and specific data descriptions were unclear; 3. The case group suffered not only from periodontitis but also from other diseases; 4. The study did not set up a control group; and 5. The study was conference literature.

### Data extraction

Data were independently extracted from the evaluated information and cross-checked by two reviewers (WB Zhang and QH Mao). Inconsistencies were resolved by discussion or by consulting a third researcher (YN Cheng). The assessed information included the name of the first author, the date of publication, the distribution area of the study subjects, the genotype distributions, the Hardy-Weinberg balance index and the disease descriptions [8]. The quality of the literature included in the study was evaluated using the NOS scale (Newcastle-Ottawa quality assessment scale) [9]. The table was defined and selected from the case and control groups to assess the comparability between the two groups. The identification of exposure factors was based on three aspects of the score, with a literature quality score ≥ 6 representing high quality.

### Data analysis

Odds ratios (ORs) and the corresponding 95% confidence intervals (CIs) for the pooled data were used to determine the relationship between the assessed IL-13 -1112C/T SNP and susceptibility to periodontal disease.

We used five models, including an allele model (C vs T), co-dominant models (CT vs TT and CC vs TT), a dominant model (CC vs CT + TT) and a recessive model (TT vs CT + CC), and subgroup analyses were performed based on the case type. The Chi-square test and Q-test were utilized to assess the heterogeneity of the data. If *P* > 0.10 or I^2^<50%, the merged data exhibited good homogeneity, and a fixed-effects model was used for the analysis; otherwise, a random-effects model was employed. A subgroup analysis was performed based on the disease type. The software used for the aforementioned analysis was STATA12.0 (Stata Corp. LP, College Station, TX, USA). Finally, each study was removed individually for the sensitivity analysis prior to the conclusion of the study.

## Results

### Study selection and characteristics

**Fig. 1:**
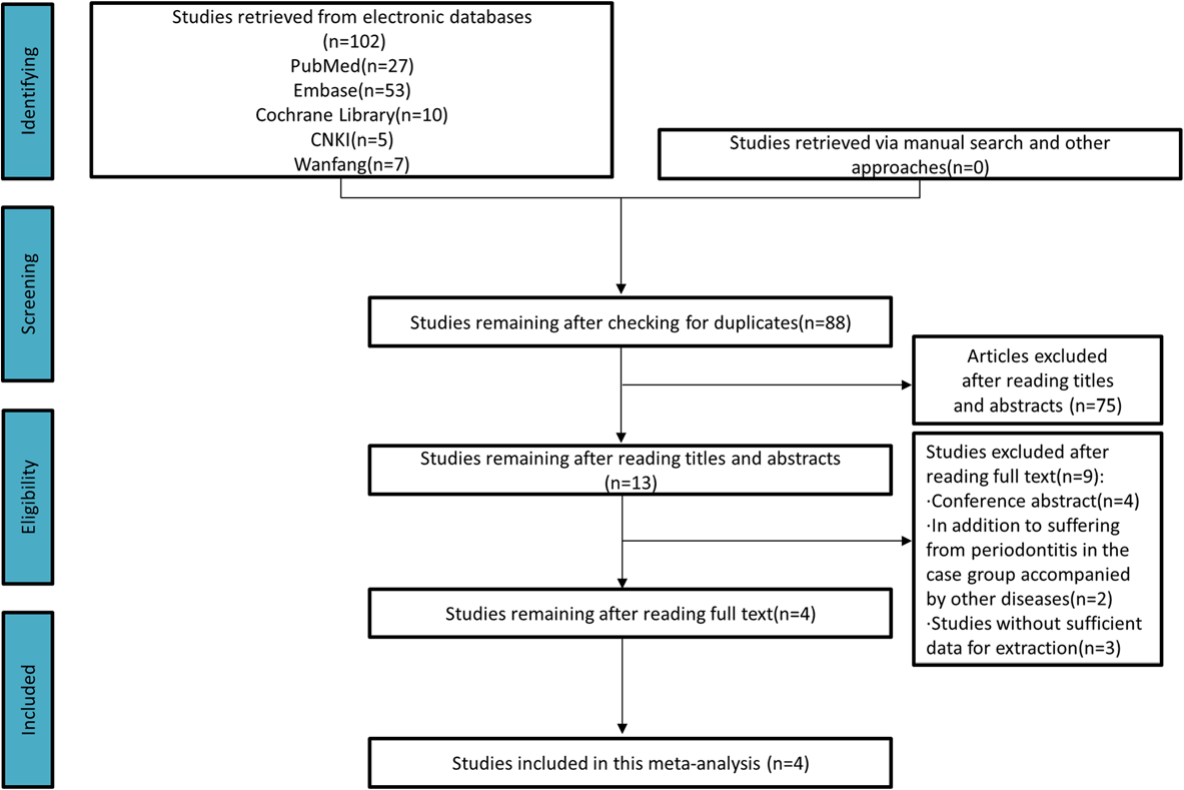
Flow chat for study retrieval and screening

In accordance with the search strategy, a total of 102 articles were retrieved, including 12 articles in Chinese and 90 articles in English. After removal of duplicate publications, 88 articles remained. Thirteen articles were obtained after titles and abstracts were reviewed and screened. After full-text screening, four articles representing five case-control studies were eventually included in the meta-analysis [10–13] (Fig. 1). One of the articles [11] investigated AgP and CP patients and could thus be regarded as two independent cases-control studies. These studies involved 671 healthy controls and 710 cases, including 592 cases in the CP group and 118 cases in the AgP group (Table 2). The results of the literature review showed that the diagnosis of periodontitis was clear with the exception of one study that did not meet the balance of the Hardy-Weinberg equilibrium (HWE). The selection of the control groups was well defined, the data were intact and the groupings were well matched. The studies were good quality and had adequate sample sizes. Thus, the overall inclusion criteria resulted in good quality studies.

**Table 2:** Characteristics of the included studies

Author	Year	Distribution area	Cases (*n*)	Controls (*n*)	Case genotype			Control genotype			HWE (*P*)	Gene detection method	Disease type	Quality Score
					CC	CT	TT	CC	CT	TT				
Gonzales	2007	Germany	58	51	19	29	10	17	29	5	0.145	PCR-RFLP	AgP	7
Wu YM1	2010	China	60	95	47	13	0	52	41	2	0.059	PCR-RFLP	AgP	6
Wu YM2	2010	China	204	95	117	84	3	52	41	2	0.059	PCR-RFLP	CP	6
Zhang YJ	2012	China	110	106	81	24	5	70	32	4	0.886	PCR-RFLP	CP	6
Chen D	2013	China	278	324	171	67	40	208	92	24	0.004	MALDI-TOF-MS	CP	6

### Meta-analysis results

#### Five types of genetic models and the relationship between IL-13-1112C/T and periodontitis

The CC vs TT, CTvsTT and TT vs CT + CC models wereused in four of the five studies included in this meta-analysis. The pooled analyses using these models indicated that the IL-13 -1112C/T polymorphism was significantly associated with susceptibility to periodontitis (CC vs TT: OR = 0.615, 95% CI = 0.395–0.957; CTvsTT: OR = 0.518, 95% CI = 0.323–0.830;and TT vs CT + CC: OR = 1.739, 95% CI = 1.130–2.676). The other two models (T vs C: OR = 1.113, 95% CI = 0.776–1.595 and CC vs CT + TT: OR = 1.255, 95% CI = 0.858–1.836) suggested that the IL-13 -1112 polymorphism was not associated with the pathogenesis of periodontitis.

#### Subgroup analysis based on type of periodontitis

**Fig. 2:**
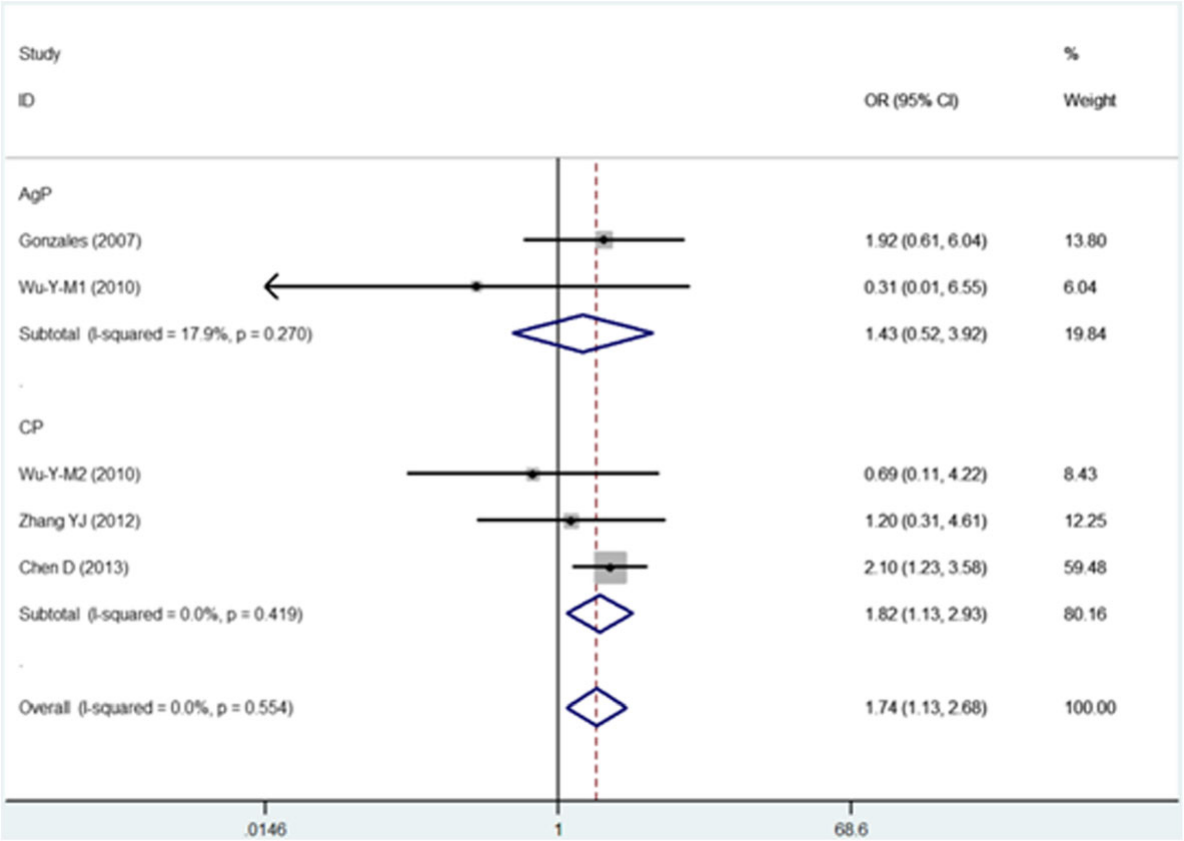
Meta-analysis for the TT vs CC + CT model in the general population and subgroups

**Fig. 3:**
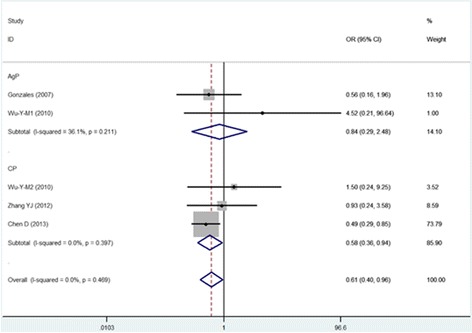
Meta-analysis for the CC vs TT model in the general population and subgroups

**Fig. 4:**
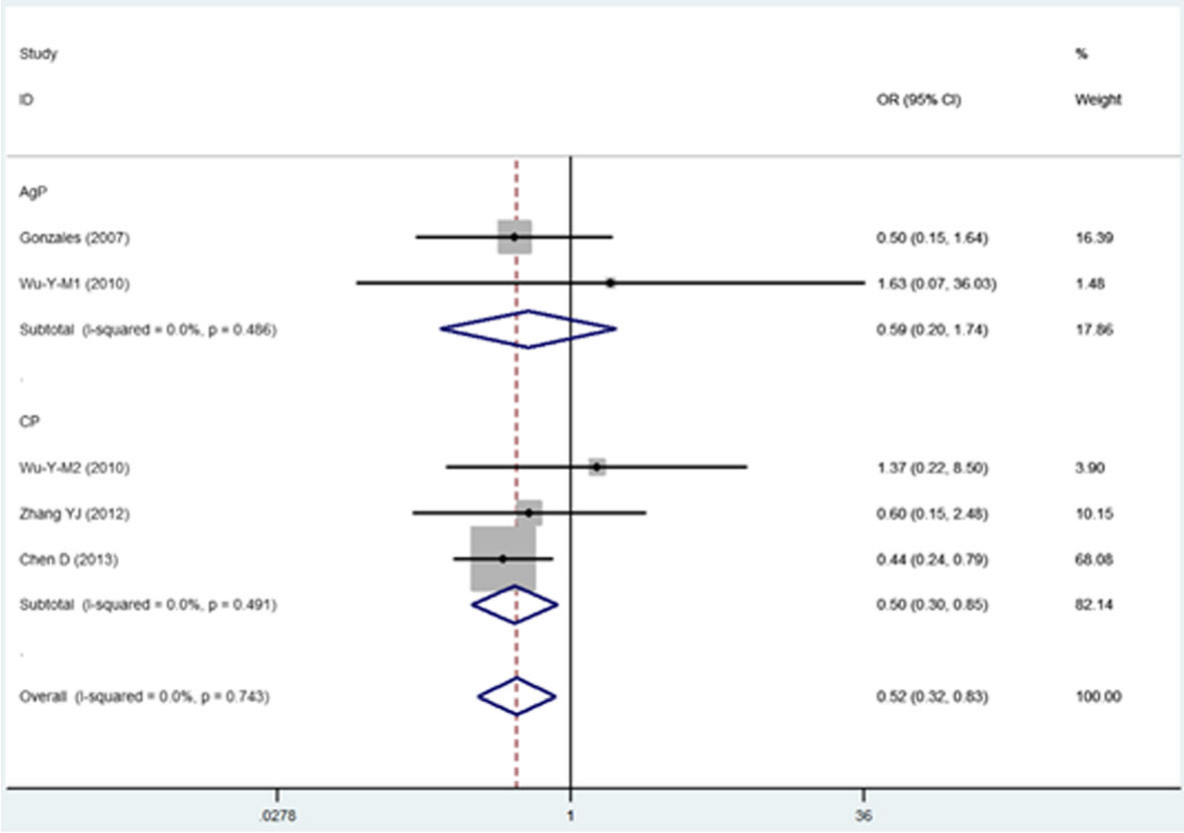
Meta-analysis for the CT vs TT model in the general population and subgroups

The subgroup analysis based on CP and AgP revealed correlations of susceptibility with IL-13 -1112. In the five-cohort analysis for the CP group, the IL-13 -1112C/T polymorphism was associated with susceptibility to CP. Significant associations were found in the CT vs TT model, the CC vs TT model and the TT vs CT + CC model (Figs. 2, 3, and 4) (TT vs CT + CC: OR = 1.816, 95% CI = 1.127–2.926; CC vs TT: OR = 0.578, 95% CI = 0.355–0.939; and CT vs TT: OR = 0.501, 95% CI = 0.296–0.848) but not in the remaining two models (T vs C: OR = 0.970, 95% CI = 0.708–1.331 and CC vs CT + TT:OR = 1.032, 95% CI = 0.800–1.331). The analysis for AgP(T vs C: OR = 1.445, 95% CI = 0.489–4.271; CC vs TT: OR = 0.840, 95% CI = 0.285–2.475; CT vs TT: OR = 0.593, 95% CI = 0.203–1.735; CC vs CT + TT:OR = 1.727, 95% CI = 0.575–5.181; and TT vs CT + CC: OR = 1.427, 95% CI = 0.520–3.921) revealed that the IL-13 -1112C/T polymorphism was not associated with AgP susceptibility. Thecorrelations between IL-13 -1112C/T and periodontitis in the different models are presented in Table 3.

**Table 3:** Overall population and subgroup analysis results for the IL-13-1112 polymorphism for different models

Model	Disease type	OR	95% CI	P	I^2^
	Periodontitis	1.113	0.776–1.595	0.012	68.9%
C vsT	CP	0.970	0.708–1.331	0.128	51.3%
	AgP	1.445	0.489–4.271	0.012	84.3%
	Periodontitis	0.615	0.395–0.957	0.469	0.0%
CC vsTT	CP	0.578	0.355–0.939	0.397	0.0%
	AgP	0.840	0.285–2.475	0.211	36.1%
	Periodontitis	0.518	0.323–0.830	0.743	0.0%
CT vsTT	CP	0.501	0.296–0.848	0.491	0.0%
	AgP	0.593	0.203–1.735	0.486	0.0%
	Periodontitis	1.255	0.858–1.836	0.050	57.8%
CC vsCT+TT	CP	1.032	0.800–1.331	0.355	3.4%
	AgP	1.727	0.575–5.181	0.043	75.6%
	Periodontitis	1.739	1.130–2.676	0.554	0.0%
TT vsCT+CC	CP	1.816	1.127–2.926	0.419	0.0%
	AgP	1.427	0.520–3.921	0.270	17.9%

### Sensitivity analysis

A sensitivity analysis for the included studies was performed by excluding each of the studies in turn. The findings from the meta-analysis did not change after each study was excluded. This result indicates that our findings are realistic and reliable.

### Publication bias analysis

Since a small number of studies was included in this report, approaches to detect publication bias would have exhibited limited efficacy; therefore, publication bias was not assessed.

## Discussion

Periodontitis is an inflammatory infectious disease that is the main cause of the loss of human teeth; its main features are gingival inflammation, periodontal pocket formation, alveolar bone resorption and tooth loosening and displacement [14]. An early sign of gingivitis is dental plaque accumulation, which with further development of inflammation may progress into periodontitis. However, plaque reactions are not consistent between individuals; some plaque accumulation occurs when gingivitis develops, followed by further development of periodontitis, whereas other individuals exhibit plaque accumulation but do not progress to periodontitis. Therefore, genetic factors play an important role in the development and progression of periodontitis. Recent evidence has suggested that individual differences in susceptibility to periodontitis are associated with genes [15, 16].

The IL-13 gene is located on chromosome 5 (5q23.31). The gene is 4.6 kb in length, contains 4 exons, 3 introns and 2 methionine initiation codons and encodes a 132-amino acid protein [17]. The non-glycosylated protein has a relative molecular weight of 12,000 Da. IL-13 is a multi-potent cytokine with various biological effects [18]. For instance, IL-13 is a key inducer of many pathological processes that are determined by class II cytokines [19]. It can regulate inflammation, mucus production, tissue remodeling and fibrosis and as a result has become a therapeutic target for many diseases, including asthma, idiopathic pulmonary fibrosis, ulcerative colitis and other IL-13 overexpression diseases [20–22].

During the process of periodontitis, type II helper T lymphocytes (TH2) secrete IL-4, IL-13 and other cytokines that are conducive to B cell humoral immunity and improve the symptoms of inflammation. These cytokines can inhibit the secretion of tumor necrosis factor-α (TNF-α) by mononuclear cells to achieve some anti-inflammatory effects and can activate transforming growth factor-β1 (TGF-β1) and thus produce fibrosis. IL-13 can affect bone resorption by affecting the bone protection factor system and can directly promote IgE and thus cytokine synthesis in humans. Studies have found that periodontitis in the gingival tissue is associated with a significant increase in IgE and that IL-13 can stimulate cells in periodontal lesions to produce and fight against periodontal pathogens that express lipopolysaccharide. Therefore, understanding the biological role of IL-13 and its gene polymorphisms is necessary for an in-depth study of periodontitis. Of the single nucleotide polymorphic loci of IL-13, the − 1112 locus was the first discovered and the most studied. In 1999, the Dutch scholar Van der Pour Kranfirst [23] confirmed that the site could be enhanced. The binding of the nucleoprotein to the promoter region increased IL-13 synthesis. Nie et al. [24] showed that the IL-13 -1112 gene polymorphism was associated with the occurrence of asthma in Caucasians; however, this site was not associated with susceptibility to asthma among Asians and African Americans. A number of studies have shown that the IL-13 -1112 gene polymorphism is associated with a variety of diseases, including type 2 diabetes, chronic obstructive pulmonary disease, allergic rhinitis, inflammatory bowel disease and colorectal cancer [25, 26]. The IL-13 -1112 gene polymorphism is associated with susceptibility to periodontitis, which has been studied in recent years.

Currently, genetic polymorphisms and periodontitis are studied using case-control studies. However, case-control studies can have insufficient sample sizes, neglect confounding factors and include multiple statistical measurements. Additionally, case-control studies themselves can demonstrate weakness and other defects [27], resulting in reduced reliability of the results. A meta-analysis [28] is a systematic quantitative analysis method for the consolidation of multiple similar studies. The advantage of this approach is that it can increase the sample content from the statistical perspective and improve the test efficiency, especially when multiple studies are included. When the results are not significant or the conclusion is inconsistent, the meta-analysis method can be closer to the real situation of the system analysis results. Meta-analysis results have been used as best evidence in health decision-making and clinical practice and have played an increasingly important role. The present study on the association between the IL-13 -1112C/T gene polymorphism and periodontitis is different. Therefore, this meta-analysis of the IL-13-1112C/T gene polymorphism and periodontitis susceptibility used case-control studies to conduct a systematic quantitative analysis of the IL-13-1112C/T gene polymorphism and susceptibility to periodontitis and to provide evidence-based medical evidence.

This study systematically collected relevant case-control studies, merged them to discuss the relevance of the site and the incidence of periodontitis and provided meta-analysis results. Five studies were included, with 2 studies conducted on AgP. Gonzales [10] suggested that the IL-13-1112 genotype and the C and T allele frequencies were not associated with susceptibility to AgP, whereas Wu et al. [11] suggested that the CC genotype of IL-13 -1112 was a risk factor for AgP compared to the CT/TT genotypes. Three studies investigated CP. Zhang et al. [12] showed that the IL-13 -1112 allele frequency and genotype frequency were independent of the CP susceptibility, which was the same as the finding of Wu et al. [11]. Chen et al. [13]. showed that the IL-13-1112 gene polymorphism might be associated with susceptibility to CP. All of the included studies had quality scores greater than 6 points, indicating that the quality of the included literature was high. To maximize the correlation between the genetic model and periodontitis, the CC vs TT, CC vs CT and TT vs CT models were used to analyze the correlation between the models. The correlation for the TT model + CC model was significant. In the subgroup analysis for the disease-based groups, the differences between the CT vs TT, CC vs TT and TT vs CT + CC models were significant in the CP group. The heterogeneity was low, and the conclusion was reliable, suggesting that the IL-13 -1112 gene polymorphism was associated with CP susceptibility. The five models in the AgP group showed no significant differences. According to the results of this study, the association of the IL-13 -1112 gene polymorphism with periodontitis may be due to differences in the disease (CP and AgP), which are presumably due to factors such as race, region, predilection, suspicious disease-causing bacteria and etiology. The IL-13-1112 gene polymorphism may play different roles in the pathogenesis of CP and AgP; thus, the same gene polymorphic sites may occur in patients with CP and AgP, underscoring the relevance of the differences that lead to different susceptibilities. Heterogeneity is one factor that affects the reliability of our results. The statistical analysis of the CC vs CT + TT model (*P* = 0.050, I^2^ = 57.8%) with the C vs T model (*P* = 0.012, I^2^ = 68.9%) suggests that *P*<0.1 or I^2^ > 50% indicates that the merged data are heterogeneous, although the sensitivity analysis failed to find the source of heterogeneity. Possible sources of heterogeneity may include the regional population, genetic testing, quantity and the research process.

This study has the following limitations. 1. The number of original studies included in this study is small. Additionally, one of the studies used MALDI-TOF-MS, whereas the others used PCR-RFLP. The sensitivity of the two methods results in a certain degree of inconsistency. The control group in another study did not meet the HWE balance; thus, the control group subjects may exhibit population bias. The above issues limit the credibility of the results of this study but also show the need for the current study. More high-quality studies should be conducted to verify the conclusions of this study. 2. Of the studies included in this meta-analysis, one case-control study investigated a German population, and four studies investigated Chinese populations. Because the study’s language limitations were English and Chinese, relevant literature on different racial groups published in other languages may exist and requires further analysis. 3. In this meta-analysis study, only two cases included patients with AgP. The number is small; therefore, studies with a larger sample size are needed to confirm the relationship between the IL-13 -1112 gene polymorphism and AgP. 4. The commonly used detection methods for publication bias are Egger’s regression method [29], the funnel regression method [30], Begg’s rank method [31] and the trim and fill method [32]. Some scholars have noted that at least five independent studies (the point of the funnel map > 5) [33] are required to obtain a persuasive result with the funnel regression method. Additionally, some scholars believe that Begg’s rank method and Egger’s regression method are less sensitive for the identification of publication bias when they are included in the study [34]. Certain risks are inherent in the identification of publication bias; for instance, publication bias may be overestimated when the study is too small. Therefore, these methods should be used with caution [35]. Since the number of studies included in this study is small, some bias is likely to exist in this study because several commonly used bias detection methods are limited in this case.

## Implications for future research

Periodontitis is a complex genetic disease, and genetic factors play important roles in its severity and progression. Joint genetics analyses are useful and enable better understand of the etiology and pathogenesis of this disease. Recently, the study of IL-13 gene polymorphisms and their relationships with periodontitis contributed to our understanding, but the results of the current study were not clear and some issues did exist. Taking into account the importance of IL-13 in the development of periodontitis, a large sample case-control study must be developed to examine the involvement of genetic and environmental interactions and other factors to more reliable study the relationships between IL-13 gene polymorphisms and periodontitis. At the same time, periodontitis is not a single gene disease but instead is a multi-gene-related disease. IL-13 gene polymorphisms may play roles in periodontitis by affecting other genes that are undetected. Therefore, in addition to the need for an in-depth study of a single gene, there is also a need for a comprehensive study of a number of genetic loci to expand the sample size and better reveal the roles of IL-13 gene polymorphisms in periodontitis development. Identification of the genetic predisposing factors of periodontitis according to regulation of the host immune response at the genetic level will aid in the diagnosis, prevention and treatment of periodontitis and help maintain periodontal health.

## Conclusions

Despite these limitations, this meta-analysis showed that the IL-13 -1112 gene polymorphism might be associated with susceptibility to periodontitis. The subgroup analysis of the disease suggested that the IL-13 -1112 gene polymorphism was associated with CP susceptibility but not AgP susceptibility. Considering the importance of IL-13 in the development of periodontitis, a case-control study with a large sample size must be developed to explore the correlation between the IL-13 -1112C/T polymorphism and periodontitis.

## Abbreviations

AgP: aggressive periodontitis
CIs: confidence intervals
CNKI: China National Knowledge Infrastructure
CP: chronic periodontitis
IL-13: Interleukin-13
ORs: Odds ratios
SNPs: Single nucleotide polymorphisms
TH2: type 2 helper T
